# Metagenome of the Siberian Underground Water Reservoir

**DOI:** 10.1128/genomeA.01317-17

**Published:** 2017-11-22

**Authors:** Vitaly V. Kadnikov, Yulia A. Frank, Andrey V. Mardanov, Alexey V. Beletsky, Olga V. Karnachuk, Nikolai V. Ravin

**Affiliations:** a Faculty of Biology, Lomonosov Moscow State University, Moscow, Russia; b Institute of Bioengineering, Research Center of Biotechnology of the Russian Academy of Sciences, Moscow, Russia; c Laboratory of Biochemistry and Molecular Biology, Tomsk State University, Tomsk, Russia

## Abstract

We report on the metagenome of a deep subsurface aquifer in the Tomsk region of Russia, sampled via an oil exploration borehole drilled to a depth of 2.8 km. Methanogenic archaea were present in the water along with members of various bacterial phyla, including *Firmicutes*, *Chloroflexi*, *Bacteroidetes*, *Ignavibacteriae*, and uncultured candidate divisions.

## GENOME ANNOUNCEMENT

The deep subsurface biosphere, one of the least studied ecosystems on Earth, contains communities of extremophilic microorganisms thriving under conditions of high temperature and pressure. Microbial communities in deep subsurface ecosystems can be diverse in composition, depending on geochemical conditions (1, 2). These ecosystems remain isolated from the surface biosphere for thousands to millions years and do not depend on the input of organic substances from the surface (3, 4). Some deep subsurface microbial communities comprise mostly chemolithoautrophic microorganisms that use abiotically produced molecular hydrogen as an energy source (5). In other subsurface ecosystems, buried organic matter supports the growth of organotrophs (6).

The thermal artesian waters of the West Siberian oil basin can be accessed via oil exploration boreholes. The aquifers are located at depths of 1 to 3 km and presumably were formed in the sedimentary rocks of the Mesozoic Era. Our previous studies revealed diverse microbial communities, the composition of which differed when different boreholes were sampled and analyzed (7, 8). Here, we report a metagenome from borehole 5P, drilled to a depth of 2.8 km near the village of Chazhemto in the Tomsk region of Russia.

The thermal water had a neutral pH (7.43 to 7.6) and a low (−304 to −338 mV) redox potential (9). Metagenomic DNA was isolated from microbial biomass collected by filtration (0.2-μm filters) using a Power Soil DNA isolation kit (MO BIO Laboratories, Inc., Carlsbad, CA, USA). The sequencing of a paired-end (2 × 250 bp) TruSeq DNA library using an Illumina HiSeq2500 platform generated 57,579,354 read pairs. Adapters were trimmed using Cutadapt version 1.14 (10). Sequencing reads were preprocessed by trimming low-quality read ends (quality score of <33) using Sickle version 1.33 (https://github.com/najoshi/sickle). Trimmed reads were merged with FLASH v1.2.11 (11). Resulting merged and unmerged reads (about 16.9 Gbp in total) were *de novo* assembled using metaSPAdes version 3.7.1 (12) into 185,598 contigs longer than 500 bp.

Contigs comprising partial or complete 16S rRNA gene sequences were identified using CheckM (13). The RDP Classifier (14) and a BLASTN search against the NCBI nonredundant (NR) database were used for the taxonomic assignment of these sequences. Consistently with results of 16S rRNA profiling (9), we found methanogenic *Archaea* (*Methanosaeta*, *Methanothermobacter*, *Methanobacterium*, and *Methanomassiliicoccus*) and various groups of bacteria including representatives of the phyla *Firmicutes*, *Chloroflexi*, *Ignavibacteriae*, *Bacteroidetes*, and uncultured candidate divisions. Binning of contigs using CONCOCT (15) allowed us to obtain genome bins representing composite genomes of dominant microbial species with more than 90% completeness, as estimated by CheckM (13). The obtained genomic data will be used to describe the phylogenetic and functional diversity of the aquifer and to predict the biogeochemical reactions in the deep subsurface biosphere that may be performed by uncultured prokaryotes.

### Accession number(s).

The sequences obtained in this project have been deposited in the NCBI Sequence Read Archive under the accession number SRR6186653.

## References

[1] Jørgensen BB 2012 Shrinking majority of the deep biosphere. Proc Natl Acad Sci U S A 109:15976–15977. doi:10.1073/pnas.1213639109. 23012471 PMC3479554

[2] Gihring TM, Moser DP, Lin L-H, Davidson M, Onstott TC, Morgan L, Milleson M, Kieft TL, Trimarco E, Balkwill DL, Dollhopf ME 2006 The distribution of microbial taxa in the subsurface water of the Kalahari Shield, South Africa. Geomicrobiol J 23:415–430. doi:10.1080/01490450600875696.

[3] Edwards KJ, Becker K, Colwell F 2012 The deep, dark energy biosphere: intraterrestrial life on earth. Annu Rev Earth Planet Sci 40:551–568. doi:10.1146/annurev-earth-042711-105500.

[4] Lever MA, Rogers KL, Lloyd KG, Overmann J, Schink B, Thauer RK, Hoehler TM, Jørgensen BB 2015 Life under extreme energy limitation: a synthesis of laboratory- and field-based investigations. FEMS Microbiol Rev 39:688–728. doi:10.1093/femsre/fuv020. 25994609

[5] Takai K, Gamo T, Tsunogai U, Nakayama N, Hirayama H, Nealson KH, Horikoshi K 2004 Geochemical and microbiological evidence for a hydrogen-based, hyperthermophilic subsurface lithoautotrophic microbial ecosystem (HyperSLiME) beneath an active deep-sea hydrothermal field. Extremophiles 8:269–282. doi:10.1007/s00792-004-0386-3. 15309563

[6] Orphan VJ, Goffredi SK, DeLong EF, Boles JR 2003 Geochemical influence on diversity and microbial processes in high temperature oil reservoirs. Geomicrobiol J 20:295–311. doi:10.1080/01490450303898.

[7] Frank YA, Kadnikov VV, Gavrilov SN, Banks D, Gerasimchuk AL, Podosokorskaya OA, Merkel AY, Chernyh NA, Mardanov AV, Ravin NV, Karnachuk OV, Bonch-Osmolovskaya EA 2016 Stable and variable parts of microbial community in Siberian deep subsurface thermal aquifer system revealed in a long-term monitoring study. Front Microbiol 7:2101. doi:10.3389/fmicb.2016.02101. 28082967 PMC5187383

[8] Kadnikov VV, Frank YA, Mardanov AV, Beletskii AV, Ivasenko DA, Pimenov NV, Karnachuk OV, Ravin NV 2017 Uncultured bacteria and methanogenic archaea predominate in the microbial community of Western Siberian deep subsurface aquifer. Microbiology 86:412–415. doi:10.1134/S0026261717030079.

[9] Kadnikov VV, Frank YA, Mardanov AV, Beletsky AV, Ivasenko DA, Pimenov NV, Karnachuk OV, Ravin NV 2017 Variability of the composition of the microbial community of the deep subsurface thermal aquifer in Western Siberia. Microbiology 86:765–772.

[10] Martin M 2011 Cutadapt removes adapter sequences from high-throughput sequencing reads. EMBnet j 17:10–12. doi:10.14806/ej.17.1.200.

[11] Magoč T, Salzberg SL 2011 FLASH: fast length adjustment of short reads to improve genome assemblies. Bioinformatics 27:2957–2963. doi:10.1093/bioinformatics/btr507. 21903629 PMC3198573

[12] Bankevich A, Nurk S, Antipov D, Gurevich AA, Dvorkin M, Kulikov AS, Lesin VM, Nikolenko SI, Pham S, Prjibelski AD, Pyshkin AV, Sirotkin AV, Vyahhi N, Tesler G, Alekseyev MA, Pevzner PA 2012 SPAdes: a new genome assembly algorithm and its applications to single-cell sequencing. J Comput Biol 19:455–477. doi:10.1089/cmb.2012.0021. 22506599 PMC3342519

[13] Parks DH, Imelfort M, Skennerton CT, Hugenholtz P, Tyson GW 2015 CheckM: assessing the quality of microbial genomes recovered from isolates, single cells, and metagenomes. Genome Res 25:1043–1055. doi:10.1101/gr.186072.114. 25977477 PMC4484387

[14] Wang Q, Garrity GM, Tiedje JM, Cole JR 2007 Naïve Bayesian classifier for rapid assignment of rRNA sequences into the new bacterial taxonomy. Appl Environ Microbiol 73:5261–5267. doi:10.1128/AEM.00062-07. 17586664 PMC1950982

[15] Alneberg J, Bjarnason BS, de Bruijn I, Schirmer M, Quick J, Ijaz UZ, Lahti L, Loman NJ, Andersson AF, Quince C 2014 Binning metagenomic contigs by coverage and composition. Nat Methods 11:1144–1146. 25218180 10.1038/nmeth.3103

